# Age dependency of plasma vitamin B12 status markers in Dutch children and adolescents

**DOI:** 10.1038/s41390-021-01372-2

**Published:** 2021-02-11

**Authors:** M. Rebecca Heiner-Fokkema, Ineke J. Riphagen, Nicole S. Wiersema, Jelmer J. van Zanden, Jenny E. Kootstra-Ros, Tineke H. Pinxterhuis, H. Louise Hooimeijer, Francjan J. van Spronsen, Anneke C. Muller Kobold, Wilhelmina H. A. de Jong

**Affiliations:** 1 Department of Laboratory Medicine, University of Groningen, University Medical Center Groningen, Groningen, The Netherlands; 2 Department of Clinical Chemistry, Certe Groningen, Martini Hospital Groningen, Groningen, The Netherlands; 3 Beatrix Children’s Hospital, University of Groningen, University Medical Center Groningen, Groningen, The Netherlands; 4 Present Address: Department of Cardiology, Thoraxcentrum Twente, Medisch Spectrum Twente, Enschede, The Netherlands; 5 Present Address: Saltro Diagnostic Centre, Utrecht, The Netherlands

## Abstract

**Background:**

Vitamin B12 deficiency in children may be associated with (severe) neurological manifestations, therefore recognition is important. Diagnosing vitamin B12 deficiency in children is challenging. This study aimed to investigate plasma methylmalonic acid, holotranscobalamin, and total cobalamin in children 0–18 years of age and to estimate age-dependent reference intervals.

**Methods:**

Plasma vitamin B12 markers were measured in collected plasma samples of 170 children 0–18 years visiting a local primary care laboratory. All had within-reference hemoglobin and MCV values. Pediatric plasma vitamin B12 biomarkers were measured and reference values were derived thereof.

**Results:**

Plasma methylmalonic acid was higher in young children, in particular between 1 and 6 months of age; total cobalamin and holotranscobalamin were highest from 0.5 to 4 years and decreased till 10 years of age. Plasma holotranscobalamin was highly correlated with plasma total cobalamin; their ratio was independent of age. Plasma methylmalonic acid was slightly more related to total cobalamin than to holotranscobalamin. A large proportion of mainly young children would be misclassified when adult references are applied.

**Conclusions:**

Pediatric reference values for cobalamin markers are necessary to allow for early recognition and monitoring of children suspect of (clinical) cobalamin deficiency.

**Impact:**

- We analyzed three plasma vitamin B12 status markers, i.e., total cobalamin, holotranscobalamin, and methylmalonic acid, in the plasma of 170 children 0–18 years of age and were able to derive reference intervals thereof. Recognition of vitamin B12 deficiency in children is important but challenging as pediatric reference intervals for plasma vitamin B12 status markers, particularly plasma holotranscobalamin, are not well described. We think that our results may help early recognition and monitoring of children suspect of (clinical) vitamin B12 deficiency.

## Introduction

Vitamin B12 (cobalamin (Cbl)) is an indispensable water-soluble vitamin that acts as cofactor for the two enzymes methylmalonyl-CoA mutase and methionine synthase. Cbl is important, e.g., for cell growth and development of the central nervous system.^1–3^ Cbl deficiency seems to occur frequently in young children, in particular in those born to mothers with low maternal Cbl status.^3^ Early recognition and treatment of Cbl deficiency is essential for prevention of irreversible neurological damage. In children, irritability, hypotonia, developmental delay, failure to thrive, fatigue, and motor disturbances may be prominent and life threatening.^4–7^ Diagnosing Cbl deficiency, however, remains challenging. First, clinical symptoms of Cbl deficiency are non-specific. Classical symptoms include fatigue and (megaloblastic) anemia. However, both in adults and children, neurological symptoms (i.e., motor, sensory and cognitive impairments, mood changes, abnormal balance and reflexes, memory loss, stupor, and psychosis) and fatigue can occur before anemia develops and can even exist without hematological symptoms.^1,8^ Second, there is no gold standard for assessment of Cbl deficiency.^1,8,9^ A commonly applied biomarker is plasma total Cbl concentration, representing Cbl bound to its transport proteins, i.e., haptocorrin (HC; ~80%) and transcobalamin (TC; ~20%). Total Cbl concentrations are, however, affected by other factors besides Cbl status, including HC deficiency and inflammation/infection,^8–11^ complicating its diagnostic value. A more recently introduced marker, i.e., holotranscobalamin (holoTC), represents the fraction of circulating Cbl bound to TC only. This fraction is taken up by cells and is therefore referred to as active B12. Its diagnostic value is promising,^3^ but data in children are scarce.^12–14^ Plasma methylmalonic acid (MMA) and total plasma homocysteine are so-called functional markers, reflecting the amount of intra-cellular Cbl available for the two known Cbl-dependent enzymes in human, i.e., methylmalonyl-CoA mutase and methionine synthase, respectively. Their concentrations increase when insufficient Cbl is available. An elevated total plasma homocysteine concentration is less specific than plasma MMA for diagnosing Cbl deficiency, as it is also dependent on, e.g., adequate folate and vitamin B6 status.^8,10^

It is known that individuals with Cbl deficiency may express variable abnormalities in plasma vitamin B12 status markers. Within-reference total Cbl or holoTC concentrations together with elevated concentrations of either plasma MMA or total plasma homocysteine is not uncommon.^8,10^ This, in combination with non-specific symptoms and lack of a gold standard, makes diagnosis of Cbl deficiency particularly challenging. Many studies on biochemical markers have been performed in adults.^3^ Plasma vitamin B12 status markers, and in particular holoTC concentrations, in children are, however, less well described. This study therefore aimed to study plasma MMA, holoTC, and total Cbl in children and adolescents aged 0–18 years and to derive age-dependent reference intervals.

## Participants and methods

### Participants and samples

In this study, we included samples from children who visited a local primary care laboratory (CERTE, Location Martini Hospital Groningen, The Netherlands) for diagnostic or monitoring purposes. To prevent inclusion of individuals with overt Cbl deficiency, children were included only when hemoglobin (Hb) and mean corpuscular volume (MCV) were determined and within local reference limits, i.e., Hb >6.0 mmol/L (0–5 years) and >6.5 mmol/L (≥6 years), and MCV value <120 fL (0–1 years) and <98 fL (≥1 year). It was aimed to include at least 160 participants comprising 20 children in each age range: 0–1 month, 1–6 months, 6–12 months, 13–23 months, 2–4 years, 5–9 years, 10–14 years, and 15–18 years. Individuals of whom a lithium-heparin blood sample was available for additional analyses were eligible for inclusion. The Medical Ethical Committee of the University Medical Center Groningen confirmed that the Medical Research Involving Human Subjects Act did not apply, rendering official study approval unnecessary. The study protocol was performed in accordance with the Declaration of Helsinki and approved for waived consent as it concerned anonymous data and measurements in blood left-over materials (residue) without invasive procedures.

### Sample analyses

Before selection, the Li-heparin plasma samples were stored up to 6 days in the refrigerator (4 °C). Plasma total Cbl and MMA^15^ and holoTC (Manual Elecsys active B12, Roche Diagnostics) are considered stable under the applied conditions. In literature, MMA concentrations are often measured in serum. Serum and Li-heparin MMA results showed no significant bias during validation procedures. After selection, samples were anonymized and stored at −80 °C until analysis. Plasma MMA concentrations were analyzed using an online Solid Phase Extraction procedure (Symbiosis, Spark Holland) combined with liquid chromatography tandem mass spectrometry (Waters Quattro Premier XE, Waters Corporation) in batches over a 6-week period. Total Cbl and holoTC concentrations were analyzed on a Cobas 6000ce analyzer (Roche Diagnostics, Almere, The Netherlands) in batches over a 4-week period. Performance characteristics of these methods are shown in Supplementary Table. Plasma creatinine was in addition measured with an enzyme assay on Cobas 6000ce, to be able to correct for abnormal kidney function.

### Statistical analyses

Statistical analyses were performed using Excel 2010 (Microsoft), Analyse-it (Analyse-it Software, Ltd, Leeds, UK), and SPSS 23 (IBM Corporation, Chicago, IL). For further statistical analysis, the participants were divided into 6 age groups, i.e., neonates (0–1 months), infants (1–24 months), toddlers (1–2 years), preschoolers (3–5 years), school-aged children (6–12 years), and adolescents (13–18 years). Outliers were identified and defined as value >3× interquartile range (IQR) outside IQR boundaries for each age group.

After exclusion of the outliers, the median and range were determined for age, and the mean and 95% references intervals (as based on 2.5 and 97.5% percentiles) were calculated for plasma MMA, holoTC, and total Cbl concentrations and the ratio holoTC/total Cbl for each age group. Moreover, the number and percentage of children with an abnormal biomarker concentration was calculated when applying local reference intervals for adults, i.e., 90–340 nmol/L for MMA, 33–247 pmol/L for holoTC, and 145–450 pmol/L for total Cbl. Correlations were assessed by Spearman correlations. Kruskal–Wallis at *p* < 0.05 (age groups) and post hoc Wilcoxon–Mann–Whitney *U* tests (sex and age groups) were applied to test differences between age groups. *p* values were corrected for multi-comparisons according to Bonferroni; as 15 comparisons were performed to test all age groups against each other, *p* < 0.0033 was considered significant. Stepwise multiple linear regression analyses after log transformation of the variables were applied to investigate main determinants of MMA, holoTC, and total Cbl in the study group and to correct the relations between MMA, holoTC, and total Cbl for the variable age effects. Variables included in each model were MMA, holoTC, total Cbl, creatinine, Hb, MCV, age, and sex.

## Results

### Study group

**Fig. 1:**
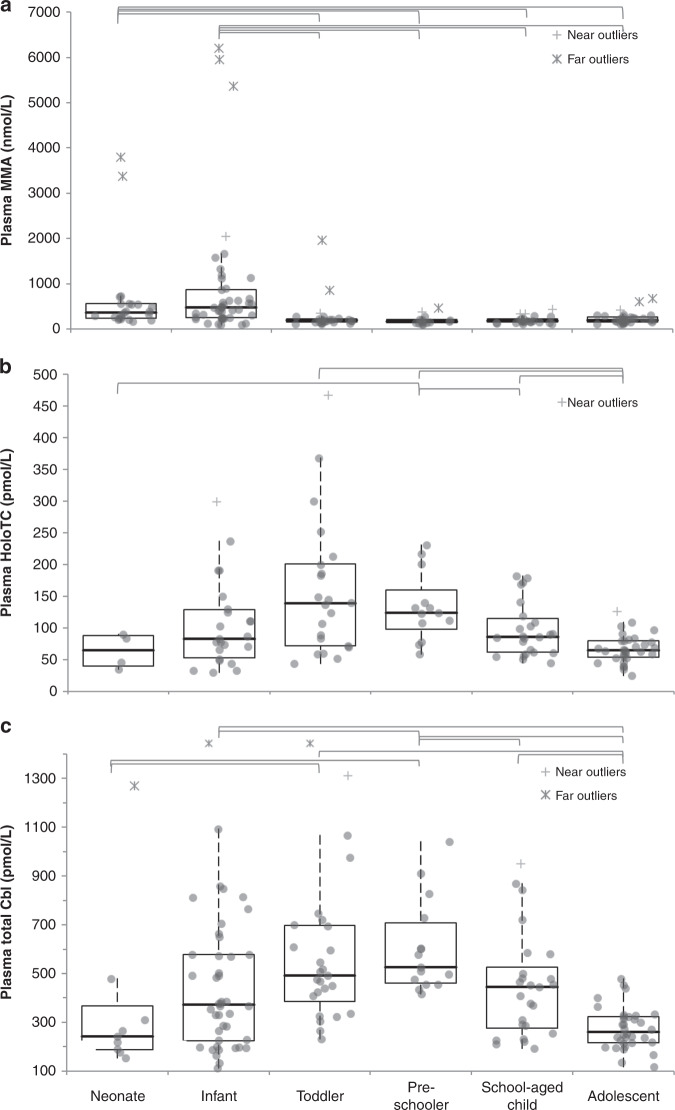
Plasma vitamin B12 status markers in six age groups. Box and whiskers plots of plasma MMA, holoTC, and total Cbl of six age groups, see Table 1 (main text). Outliers, defined as value >3× interquartile range (IQR) outside IQR boundaries, are depicted with an asterisk. Age groups with a significant biomarker concentration differences (*p* < 0.0033, corrected for Bonferroni) are connected with lines.

Samples of a total of 170 children were obtained, see Table 1. Unfortunately, analyses were not possible for 1 (MMA), 16 (creatinine), 18 (total Cbl), and 48 (holoTC) participants due to low sample volumes, in particular in the neonate and infant samples. In addition, the plasma volumes were too low to allow dilution of 5 samples with a holoTC concentration >150 pmol/L (1 neonate, 2 infants, and 1 toddler) and 2 samples with a total Cbl concentration >1476 pmol/L (1 infant and 1 toddler). We found 10 extreme outliers for MMA and 3 for total Cbl (see Fig. 1). In addition, we found 1 extreme outlier for the holoTC/total Cbl ratio. Characteristics and (estimated) reference intervals for plasma Cbl biomarkers per age category are presented in Table 1. Table 1 also shows the percentage of children who would have had an abnormal biomarker concentration when applying adult reference values. Significant age-dependent differences were apparent for MMA, holoTC, and total Cbl (see Table 1 and Fig. 1). The ratio of holoTC/total Cbl did not differ between the age groups (Table 1). Neonates and infants (groups 1–2) had significantly different plasma MMA compared to the other groups (groups 3–6). For holoTC, only significant differences existed for adolescents (6) vs toddlers, preschoolers, and school-aged children (3–5) and for preschoolers (4) vs neonates and school-aged children (1, 5). For total Cbl, significant differences existed between neonates (1) vs toddlers and preschoolers (3, 4), infants (2) vs school preschoolers and adolescents (4, 6), preschoolers (4) vs school-aged children and adolescents (5, 6) and adolescents (6) vs toddlers and school-aged children (3, 5). Significant differences between age groups are also presented in Fig. 1.

**Table 1:** Characteristics and reference intervals for plasma vitamin B12 status markers per age group.

Age category	Neonates (0–1 months)	Infants (1–12 months)	Toddlers (1–2 yrs)	Preschooler (3–5 yrs)	School-aged children (6–12 yrs)	Adolescents (13–18 yrs)	Kruskal–Wallis *p*
*N*	24	45	26	15	26	34	
Age (years)	0.008 (0.00–0.06)	0.29 (0.09–1.00)	1.64 (1.01–2.97)	3.7 (3.1–5.0)	9.1 (6.1–12.3)	15.9 (13.0–17.8)	
Gender (M/F)	16/8	21/24	16/10	8/7	13/13	12/22	
Plasma MMA (nmol/L)	343 (182–723)^a^	429 (104–1666)^a^	187 (111–315)^a^	160 (98–345)^a^	183 (117–378)	197 (112–348)^a^	*p* < 0.0001
*N* (%) <90 nmol/L	0 (0%)	0 (0%)	0 (0%)	0 (0%)	0 (0%)	0 (0%)	
*N* (%) >340 nmol/L (adult ref values)	13 (57%)	29 (64%)	3 (12%)	2 (13%)	2 (8%)	3 (9%)	
Plasma holoTC (pmol/L)	65.1 (n.a.)^a^	83.1 (31.7–264.7)^a^	138.5 (48.0–414.7)^a^	124.3 (63.6–226.4)^a^	86.3 (48.1–180.2)^a^	65.0 (32.6–113.3)^a^	*p* < 0.0001
*N* (%) <33 pmol/L	0 (0%)	1 (4%)	0 (0%)	0 (0%)	0 (0%)	1 (3%)	
*N* (%) >247 pmol/L (adult ref values)	1 (25%)	4 (17%)	5 (23%)	0 (0%)	0 (0%)	0 (0%)	
Plasma total Cbl (pmol/L)	231 (158–450)^a^	372 (134–858)^a^	491 (252–1165)^a^	525 (423–996)	447 (203–901)^a^	261 (133–458)	*p* < 0.0001
*N* (%) <145 pmol/L	0 (0%)	2 (5%)	0 (0%)	0 (0%)	0 (0%)	2 (6%)	
*N* (%) >450 pmol/L (adult ref values)	2 (22%)	18 (43%)	17 (68%)	13 (87%)	12 (48%)	2 (6%)	
Plasma holoTC/total Cbl	0.31 (n.a.)^a^	0.23 (0.12–0.52)^a,b^	0.24 (0.10–0.64)^a^	0.21 (0.11–0.40)^a^	0.21 (0.10–0.41)^a^	0.23 (0.13–0.41)^a^	n.s.

Data represent median (range) for age and median (95% reference interval) for plasma vitamin B12 status markers.

*MMA* methylmalonic acid, *HoloTC* holotranscobalamin, *Cbl* cobalamin, *n.a.* not available, *n.s.* not significant.

^a^Number of samples included lower than presented in second row due to low sample volume and for calculation of means and 95% reference ranged also due to exclusion of extreme outliers (see Fig. 1).

^b^There was one extreme outlier for holoTC/total Cbl ratio in the group of infants with a value of 0.94.

### Correlation and (multivariable) regression analyses

**Fig. 2:**
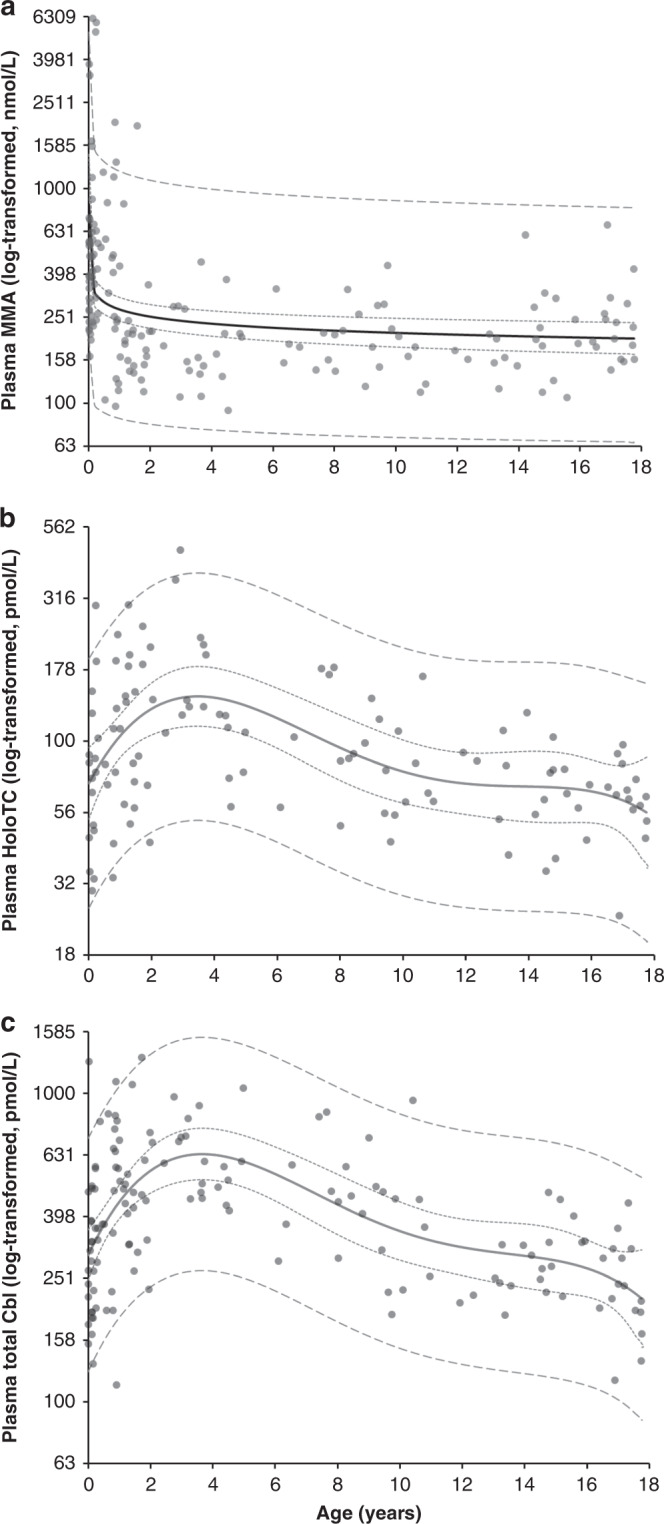
Relations between (log-scaled) plasma vitamin B12 status markers and age. Concentrations of plasma MMA (**a**), holoTC (**b**), and total Cbl (**c**) were log-transformed to produce 95% reference curves. The scales were log-transformed, but for clearance, concentrations are notated as non-transformed concentrations. Log plasma MMA was best described using a power fitted line (*R*^2^ = 0.189, *p* < 0.0001), log plasma holoTC (*R*^2^ = 0.245, *p* < 0.0001), and log total Cbl (*R*^2^ = 0.330, *p* < 0.0001)) were best described using polynomial (quartic) fitted lines.

Plasma MMA was significantly related to age, creatinine, MCV, holoTC, and total Cbl but not to Hb and sex (Table 2). A sufficient multivariable linear regression model could only be obtained after removal of the extreme outliers for MMA. Plasma MMA remained significantly related to age (Standardized Beta = −0.47, *p* < 0.0005) and total Cbl (St Beta = −0.23, *p* = 0.005). These variables explained 30% of the total variation in plasma MMA concentrations (*n* = 110). HoloTC was significantly correlated with age, creatinine, MCV, MMA, and total Cbl but not with Hb and sex (Table 2). Only total Cbl remained significantly related to holoTC in multivariable linear regression analysis (St Beta = 0.65, *p* < 0.0005), explaining 43% of the total variation in plasma total Cbl concentrations (*n* = 116). Total Cbl concentrations were significantly related to creatinine, Hb, MCV, MMA, and holoTC but not to age and sex (Table 2). Only holoTC (St Beta = 0.55, *p* < 0.0005) and MCV (St Beta = −0.31, *p* < 0.0005) remained significantly related to total Cbl in multiple linear regression analysis, explaining 51% of the total variation in total Cbl concentrations (*n* = 116). Relations between plasma vitamin B12 markers and age are presented in Fig. 2. The best fit was obtained after log-transformation of the variables. The various relations between plasma total Cbl, plasma holoTC, and plasma MMA are presented in Supplemental Fig. 1.

**Table 2:** Spearman correlation matrix.

	Plasma MMA	Plasma HoloTC	Plasma total Cbl
Plasma MMA	—	*r* = −0.21, *p* = 0.025	*r* = −0.22, *p* = 0.006
Plasma HoloTC	*r* = −0.21, *p* = 0.025	—	*r* = 0.65, *p* < 0.0005
Plasma total Cbl	*r* = −0.22, *p* = 0.006	*r* = 0.65, *p* < 0.0005	
Age	*r* = −0.48, *p* < 0.0005	*r* = −0.27, *p* < 0.0005	n.s.
Gender	n.s.	n.s.	n.s.
Hb	n.s.	n.s.	*r* = −0.18, *p* = 0.027
MCV	*r* = 0.31, *p* < 0.0005	*r* = −0.36, *p* < 0.0005	*r* = −0.45, *p* < 0.0005
Plasma creatinine	*r* = −0.13, *p* = 0.025	*r* = −0.29, *p* = 0.002	*r* = −0.29, *p* < 0.0005

*MMA* methylmalonic acid, *HoloTC* holotranscobalamin, *Cbl* cobalamin.

## Discussion

This study investigated plasma MMA, holoTC, and total Cbl concentrations in children aged 0–18 years who visited a primary care laboratory, and age-dependent reference intervals were derived thereof. All vitamin B12 status markers were significantly related to age. Plasma MMA was highest in young children, in particular between 1 and 6 months of age, whereas both total Cbl and holoTC were highest from approximately 6 months till 4 years of age and tended to slightly decrease until 10 years of age. Plasma holoTC was correlated to plasma total Cbl; their ratio was independent of age. In our population, plasma MMA was slightly more significantly related to total Cbl than to holoTC.

In our study population of 170 children, we measured three common Cbl status markers, including plasma holoTC. Recognizing Cbl deficiency is important, as in young children it may be associated with irritability, hypotonia, developmental delay, failure to thrive, fatigue, and motor disturbances.^4–7^ Symptoms can improve after Cbl supplementation^4,16^ but may remain for long periods when Cbl deficiency is unrecognized.^7^ Recognition of elevated MMA is also important for identification of genetic MMA acidurias, including inborn errors of Cbl metabolism and transport. Early treatment is mandatory for most of these disorders to prevent severe and permanent neurological damage and renal failure.^17^ Cbl status markers and specialized metabolic and genetic testing are often required to reach a final diagnosis, as reviewed by Hannibal et al.^3^ In some countries, certain defects are included in the national neonatal screening program, but screening can be complicated by false positive results due to low Cbl status, necessitating reference intervals for Cbl status biomarkers.^3^ The large number of children with abnormal metabolite concentrations when applying adult reference values, as shown in Table 1, further illustrates the need for well-established reference intervals in children.

In our study, plasma MMA was particularly high in children aged 1–6 months and remained relatively stable after the first year, which is in line with results of previous studies.^2,12,13,18,19^ Indeed, 30% of the children around 4 months of age had MMA levels >1000 nmol/L. Higher MMA concentrations in young children compared to other age groups seems to be particularly dependent on maternal nutritional factors during pregnancy and breastfeeding.^12,19^ Children who are only breastfed have significantly lower Cbl status, i.e., higher plasma MMA and lower total Cbl, compared to formula-fed children,^4,16^ in particular, but not exclusively, when their mothers consume a macrobiotic diet.^20^ Of note, 10 individuals were identified with largely elevated MMA concentrations. The reason for this could only be speculated as the samples were not traceable to the subject and could not further be investigated. At least one subject may have had Cbl deficiency, as plasma total Cbl and holoTC were also low. Other causes may include an elevated synthesis from propionic acid produced by the anaerobic gut flora, kidney immaturity, recreational nitrous oxide abuse (though not likely at very young age), sporadic (heterozygous) mutations in genes coding for 3-hydroxy-isobutyryl-CoA hydrolase and acyl-CoA synthetase family member 3,^21^ or inborn errors of MMA or Cbl metabolism.^3^ Unexplained transient or persistent high MMA, without evidence of Cbl insufficiency or other obvious cause, is a phenomenon more often found, further emphasizing the difficulties in diagnosing Cbl deficiency.^10^ In young children, diagnosing Cbl deficiency is probably even more difficult, as plasma MMA has a different relation with age compared to the other two plasma vitamin B12 status markers (Figs. 1 and 2).

Data about plasma holoTC concentrations in young children are scarce. In our study, holoTC was highly correlated to plasma total Cbl. Both total Cbl and holoTC were highest from 6 months till 4 years of age and tended to decrease thereafter until 10 years of age. Bailey et al.^22^ described a somewhat similar pattern with highest concentrations in children aged 1–9 years. Concentrations decreased up to 14 years of age to remain stable thereafter.^22^ HoloTC concentrations were similar to those found by others at 9 months of age^13^ and at 9 and 17 years of age.^23^ However, others found that holoTC decreased in the first 4–6 months of age from 90 pmol/L in neonates to 36 pmol/L in breastfed and 75 pmol/L in non-breastfed children in one study^12^ and from 201 pmol/L in neonates to 60 pmol/L in breastfed children in another study.^13^ Total Cbl concentrations were also decreased in human milk at 4 months,^13^ suggesting (subclinical) Cbl deficiency, particularly in breastfed children. Indeed, notwithstanding the fact that most studies showed a positive effect of exclusively breastfeeding on infant development,^24^ it was previously found that exclusively breastfed low-birth weight infants have lower Cbl status and poorer gross motor development than formula-fed low-birth weight infants.^4^ In children with biochemical Cbl deficiency, gross motor function significantly improved after Cbl treatment.^4^ Unfortunately, in our cohort, the number of samples in children up to 6 months were limited, in particular in the very young age groups. However, plasma holoTC seemed higher between 1 and 6 months compared to these previous studies, suggesting that a lower proportion of the included children had subclinical Cbl deficiency. The higher concentrations of total Cbl and holoTC between 6 months and 9 years of age may be the result of higher Cbl consumption after introduction of meat. Moreover, an increase in concentrations of the transport proteins at these ages may contribute in particularly to the increase in total Cbl as Greibe et al.^13^ showed that HC concentrations increased from 4 to 9 months of age. Concentrations of the transport proteins are not well described in children and could unfortunately not be studied in our cohort. Of interest, in the study of Greibe et al.,^13^ plasma MMA was related to plasma total Cbl but not to plasma holoTC at all time points. Also, in our study, regression analyses showed a more significant relation between total Cbl and MMA compared to holoTC, suggesting that total Cbl slightly better reflects subclinical Cbl deficiency in children than holoTC. The difference was, however, small and its possible added value needs further investigation. Plasma holoTC seems to be a more promising marker in adults; however, the value of both markers is debated as many confounders are identified or still unknown.^3,9^

The first limitation of our study is the relatively low number of children, in particular in the lowest age groups. This invalidates the accuracy of the estimated reference values. Further validation in a larger cohort is therefore highly recommended. However, as a total of 170 children were included, we were able to study the relation between the markers and age, which highlighted the importance of application of age-specific reference values. Moreover, we estimated reference intervals from children who visited a primary care laboratory for reasons unknown to us. In pediatrics, mainly due to practical and ethical reasons, it is, however, not uncommon to estimate reference intervals from children visiting a primary care laboratory.^25^ Moreover, exclusion of outliers before determining 95% reference intervals to some extent prevent inclusion of children with comorbidities affecting these biomarkers, including inflammation or cancer, which may cause total Cbl elevations.^11^ Unfortunately, our study did not measure concentrations of total plasma homocysteine and of Cbl transport proteins, i.e., HC and TC, which could have aided to the interpretation of total Cbl and holoTC. The limited amount of blood available also did not allow us to measure folate status. Folate is well known for masking the hematological symptoms of Cbl deficiency. Moreover, there are also concerns that elevated folate concentrations may worsen vitamin B12 status and increase MMA concentrations. The mechanism is not known, but it has been hypothesized that folate inactivates vitamin B12 by irreversible oxidation.^26^ Although all included children had normal Hb and MCV, (sub)clinical Cbl deficiency cannot be excluded when hematological parameters are within reference limits.^1,8^ Moreover, it may be argued that applying 95% reference intervals for nutritional markers is questionable, due to confounders even in healthy populations. Unfortunately, there is no gold standard for Cbl status,^9^ which makes it particularly difficult to determine the reference population and to establish cut-off values for optimal vitamin B12 status. Nonetheless, as plasma Cbl biomarker concentrations in our study did not in essence differ from those reported by others who studied one of the parameters in apparently healthy children, the proposed reference intervals may be considered a good starting point for further studies on this subject. Moreover, these intervals can be applied to facilitate diagnosis of clinical Cbl deficiency in children, presuming no significant biases between laboratory methods used.

In conclusion, we studied Cbl biomarkers and estimated age-dependent reference intervals for plasma MMA, holoTC, and total Cbl for children of 0–18 years of age. This may help to recognize and monitor children suspected of Cbl deficiency.

## Supplementary information

Supplementary Table

SupplementaryFigure

Supplemental Figure

## References

[1] Hunt A, Harrington D, Robinson S (2014). Vitamin B12 deficiency. BMJ.

[2] Hogeveen M (2008). Methylmalonic acid values in healthy Dutch children. Eur. J. Nutr..

[3] Hannibal, L. et al. Biomarkers and algorithms for the diagnosis of vitamin B12 deficiency. *Front. Mol. Biosci*. **3**, 27 (2016). 10.3389/fmolb.2016.00027 PMC4921487 27446930

[4] Torsvik IK, Ueland PM, Markestad T, Midttun Ø, Monsen ALB (2015). Motor development related to duration of exclusive breastfeeding, B vitamin status and B12 supplementation in infants with a birth weight between 2000-3000 g, results from a randomized intervention trials. BMC Pediatr..

[5] Graham SM, Arvela OM, Wise GA (1992). Long-term neurologic consequences of nutritional vitamin B12 deficiency in infants. J. Pediatr..

[6] Bicakci Z (2015). Growth retardation, general hypotonia, and loss of acquired neuromotor skills in the infants of mothers with cobalamin deficiency and the possible role of succinyl-CoA and glycine in the pathogenesis. Medicine.

[7] Dror DK, Allen LH (2008). Effect of vitamin B12 deficiency on neurodevelopment in infants: current knowledge and possible mechanisms. Nutr. Rev..

[8] Stabler SP (2013). Vitamin B12 deficiency. N. Engl. J. Med..

[9] Carmel R (2013). Diagnosis and management of clinical and subclinical cobalamin deficiencies: why controversies persist in the age of sensitive metabolic testing. Biochimie.

[10] Wolffenbuttel BHR, Wouters HJCM, Heiner-Fokkema MR, van der Klauw MM (2019). The many faces of cobalamin (vitamin B12) deficiency. Mayo Clin. Proc. Innov. Qual. Outcomes.

[11] Arendt JFB, Nexo E (2013). Unexpected high plasma cobalamin. Clin. Chem. Lab. Med..

[12] Hay G (2010). Maternal folate and cobalamin status predicts vitamin status in newborns and 6-month-old infants. J. Nutr..

[13] Greibe E (2013). Cobalamin and haptocorrin in human milk and cobalamin-related variables in mother and child: a 9-mo longitudinal study. Am. J. Clin. Nutr..

[14] Ok Bozkaya I, Yarali N, Kizilgün M, Ozkan S, Tunc B (2017). Relationship between the levels of holotranscobalamin and vitamin B12 in children. Indian J. Hematol. Blood Transfus..

[15] Hustad S (2012). Kinetic modeling of storage effects on biomarkers related to B vitamin status and one-carbon metabolism. Clin. Chem..

[16] Torsvik I, Ueland PM, Markestad T, Bjørke Monsen ALB (2013). Cobalamin supplementation improves motor development and regurgitations in infants: results from a randomized intervention study. Am. J. Clin. Nutr..

[17] Baumgartner MR (2014). Proposed guidelines for the diagnosis and management of methylmalonic and propionic acidemia. Orphanet J. Rare Dis..

[18] Ueland PM, Bjørke Monsen ALB (2003). Hyperhomocysteinemia and B-vitamin deficiencies in infants and children. Clin. Chem. Lab. Med..

[19] Monsen ALB, Refsum H, Markestad T, Ueland PM (2003). Cobalamin status and its biochemical markers methylmalonic acid and homocysteine in different age groups from 4 days to 19 years. Clin. Chem..

[20] Bjørke Monsen ALB, Ueland PM (2003). Homocysteine and methylmalonic acid in diagnosis and risk assessment from infancy to adolescence. Am. J. Clin. Nutr..

[21] Molloy AM (2016). A common polymorphism in HIBCH influences methylmalonic acid concentrations in blood independently of cobalamin. Am. J. Hum. Genet..

[22] Bailey D (2013). Marked biological variance in endocrine and biochemical markers in childhood: establishment of pediatric reference intervals using healthy community children from the CALIPER cohort. Clin. Chem..

[23] Caldeira-Araújo, H. et al. Homocysteine metabolism in children and adolescents: influence of age on plasma biomarkers and correspondent genotype interactions. *Nutrients*. **11**, 646 (2019). 10.3390/nu11030646 PMC6471758 30884849

[24] Kramer, M. S. & Kakuma, R. Optimal duration of exclusive breastfeeding. *Cochrane Database Syst. Rev*. **8**, CD003517 (2012). 10.1002/14651858.CD003517.pub2 PMC7154583 22895934

[25] Shaw JLV, Binesh Marvasti T, Colantonio D, Adeli K (2013). Pediatric reference intervals: challenges and recent initiatives. Crit. Rev. Clin. Lab. Sci..

[26] Miller JW, Garrod MG, Allen LH, Haan MN, Green R (2009). Metabolic evidence of vitamin B-12 deficiency, including high homocysteine and methylmalonic acid and low holotranscobalamin, is more pronounced in older adults with elevated plasma folate. Am. J. Clin. Nutr..

