# Supplementary material for: Age dependency of plasma vitamin B12 status markers in Dutch children and adolescents
Source: Pediatr Res. 2021 Feb 11;90(5):1058–64. doi: 10.1038/s41390-021-01372-2 (PMC8651506; doi:10.1038/s41390-021-01372-2)
Supplement: Supplementary file 1 — Supplementary Table [file 41390_2021_1372_MOESM1_ESM.docx]

**Supplementary Table.** Performance characteristics of plasma vitamin B12 marker assays.

| **Performance characteristics** | **Plasma total Cbl**  **(Elecsys vitamin B12 II)** | **Plasma holoTC**  **(Elecsys active B12)** | **Plasma MMA** |
| --- | --- | --- | --- |
| Principle of the method | Electrochemiluminescence immunoassay (ECLIA) | Electrochemiluminescence immunoassay (ECLIA) | Online SPE LC-MS/MS  Stable isotope dilution |
| Traceability | Traceable to the vitamin B12 WHO International Standard NIBSC (National Institute for Biological Standards) code 03/178. | Traceable to the vitamin B12 WHO International Standard NIBSC (National Institute for Biological Standards) code 03/178. | Calibration curve of methylmalonic acid (Sigma-Aldrich, Darmstadt, Germany). Recoveries at three concentrations were 94.8-101.1% |
| Limit of blanc | 36.9 pmol/L | 2.0 pmol/L | Not applicable |
| Limit of detection / quantification | 73.8 / 111 pmol/L | 3.0 / 5.0 pmol/L | Not applicable / 0.9 nmol/L |
| Linear range | 36.9 - 1476 pmol/L | 3.0 - 150 pmol/L | 0.9 - 2000 nmol/L |
| Inter-assay precision % | 2.1 - 5.2% | 2.3 - 3.2% | 4.0 - 4.4% |

**Legend to Table**

Abbreviations: MMA; methylmalonic acid, HoloTC; holotranscobalamin, Cbl; cobalamin.
