# Supplementary figures and images for: Age dependency of plasma vitamin B12 status markers in Dutch children and adolescents
Source: Pediatr Res. 2021 Feb 11;90(5):1058–64. doi: 10.1038/s41390-021-01372-2 (PMC8651506; doi:10.1038/s41390-021-01372-2)

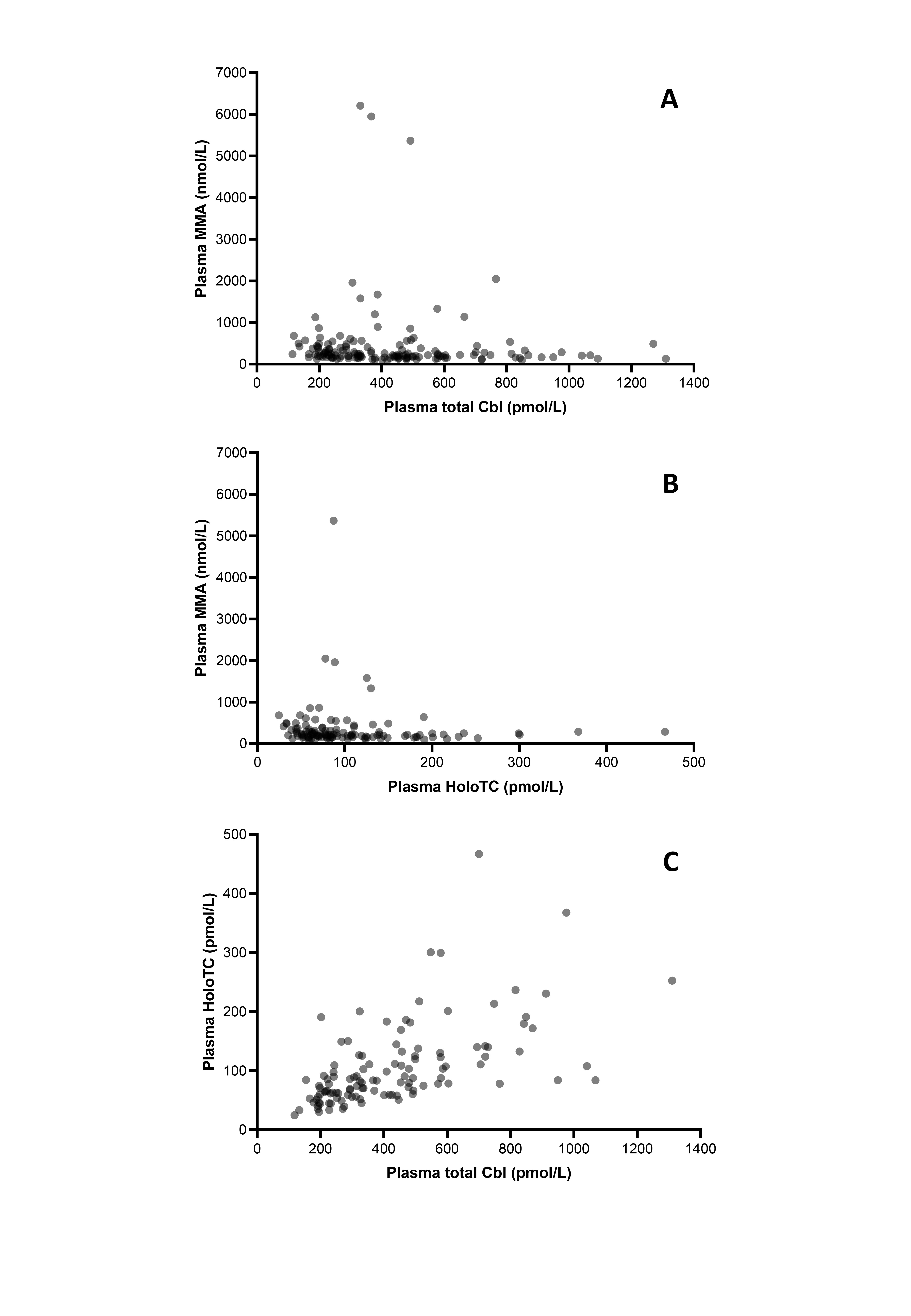

Supplement: Supplementary file 2 — SupplementaryFigure [file 41390_2021_1372_MOESM2_ESM.jpg]
