# Supplementary material for: Age dependency of plasma vitamin B12 status markers in Dutch children and adolescents
Source: Pediatr Res. 2021 Feb 11;90(5):1058–64. doi: 10.1038/s41390-021-01372-2 (PMC8651506; doi:10.1038/s41390-021-01372-2)
Supplement: Supplementary file 3 — Supplemental Figure [file 41390_2021_1372_MOESM3_ESM.docx]

Supplemental Figure. Relations between plasma vitamin B12 markers.

A: Relation between plasma total Cbl and holoTC of 117 children, Spearman r= 0.654, p<0.0005.

B: Relation between plasma holoTC and MMA of 116 children, Spearman r= -0.208, p<0.025.

C: Relation between plasma total Cbl and MMA of 149 children, Spearman r= -0.223, p<0.006.
